# Concordant morphological and molecular clines in a contact zone of the Common and Spined toad (*Bufo bufo* and *B. spinosus*) in the northwest of France

**DOI:** 10.1186/s12983-016-0184-7

**Published:** 2016-12-19

**Authors:** Jan W. Arntzen, Tania Trujillo, Roland Butôt, Klaas Vrieling, Onno Schaap, Jorge Gutiérrez-Rodríguez, Iñigo Martínez-Solano

**Affiliations:** 1 Naturalis Biodiversity Center, P.O. Box 9517, Leiden, 2300 RA The Netherlands; 2 Museo Nacional de Ciencias Naturales, CSIC, c/José Gutiérrez Abascal, 2, Madrid, 28006 Spain; 3 Plant Cluster, Institute of Biology, Sylvius Laboratory, Leiden University, P.O. BOX 9505, Leiden, 2300 RA The Netherlands; 4 Instituto de Investigación en Recursos Cinegéticos (IREC-UCLM-CSIC-JCCM), Ronda de Toledo, Ciudad Real, s/n, 13005 Spain; 5 CIBIO-InBIO, Centro de Investigação em Biodiversidade e Recursos Genéticos, Campus Agrário de Vairão, Universidade do Porto, Vairão, 4485-661 Portugal; 6 Present address: Ecology, Evolution and Development Group, Department of Wetland Ecology, Doñana Biological Station, CSIC, c/Americo Vespucio, Seville, s/n, 41092 Spain

**Keywords:** Cline analysis, Hybridization, Microsatellites, Morphology, mtDNA, SNPs

## Abstract

**Background:**

Hybrid zones are regions where individuals of two species meet and produce hybrid progeny, and are often regarded as natural laboratories to understand the process of species formation. Two microevolutionary processes can take place in hybrid zones, with opposing effects on population differentiation. Hybridization tends to produce genetic homogenization, reducing species differences, whereas the presence of mechanisms of reproductive isolation result in barriers to gene flow, maintaining or increasing differences between taxa.

**Results:**

Here we study a contact zone between two hybridizing toad species, *Bufo bufo* and *B. spinosus*, through a combination of molecular (12 polymorphic microsatellites, four nuclear and two mitochondrial SNP markers) and morphological data in a transect in the northwest of France. The results show largely concordant clines across markers, defining a narrow hybrid zone of ca. 30 km wide. Most hybrids in the centre of the contact zone are classified as F_2_ or backcrossed individuals, with no individuals assigned to the F_1_ hybrid class.

**Conclusions:**

We discuss the implications of these results for our understanding of the evolutionary history of these species. We anticipate that the toad contact zone here described will become an important asset in the study of hybrid zone dynamics and evolutionary biology because of its easy access and the abundance of the species involved.

**Electronic supplementary material:**

The online version of this article (doi:10.1186/s12983-016-0184-7) contains supplementary material, which is available to authorized users.

## Background

Speciation is usually accompanied by the gradual accumulation of genetic incompatibilities between diverging gene pools through time [1]. Thus, there are temporal windows during the speciation process where reproductive isolation is sufficient for species to remain differentiated while they are still capable of producing hybrids. This process of natural hybridization allows the incorporation of alleles of one species into the genetic background of another (introgression). Often, alleles introgressing most easily across species boundaries are those associated with an adaptive advantage, whereas those subject to divergent selection or related to reproductive isolation will show little or no introgression. Studying patterns of differential introgression across markers in hybrid zones thus provides a means to identify genes associated to local adaptation and reproductive isolation, which is key to understand how species boundaries persist in the face of gene flow [2]. When barriers to hybridization are incomplete, as in many secondary contact zones, a wide range of possible outcomes can be expected from an evolutionary perspective, from genetic homogenization to the maintenance of independent evolutionary trajectories following the evolution of full reproductive isolation. But it is the grey area in between these extremes, where different genomic regions are shown to introgress at varying levels, that provides the most information about the speciation process. This is the most common situation in nature and the reason why hybrid zones are regarded as natural laboratories for the study of speciation [3].

A powerful approach to characterize the outcomes of post-divergence hybridization and understand the underlying evolutionary processes is provided by cline theory. Geographically, hybrid zones can be defined as clines in characters that show different frequencies in the parental populations. Therefore, it is possible to study hybrid zones by sampling along a transect perpendicular to the contact zone and examining patterns of variation across multiple independent markers [4–6]. Several theoretical models have been proposed to explain the structure and temporal maintenance of hybrid zones, differing in the relative roles of extrinsic (environmental) and intrinsic (organismal) factors in maintaining some degree of reproductive isolation between hybridizing species. One of the best known examples are tension zones, where selection is environment independent and phenotypic and genetic characters usually show concordant, steep clines that are often narrow relative to the geographic distributions of the parental populations and the dispersal capacity of the organisms under study. Tension zones tend to remain stable through time because of incoming genetic flow from the parental populations at both ends of the contact zone and the elimination of hybrid individuals at the centre by natural selection [5, 7–10]. However, when tension zones form in areas of low population density, their position can shift through time if environmental conditions trigger concomitant changes in density [5].

Since the publication of some, now classical synthesis papers on the role of Quaternary Ice Ages in shaping current patterns of genetic diversity and structure across taxa [11, 12], there has been much progress in documenting hybrid zones in Europe from a comparative, cross-taxon perspective. This has revealed patterns of concordance in the geographic location of contact zones, which has often been interpreted in terms of a parallel evolutionary history (linked to inferred routes of post-glacial dispersal) on a shared geographic background, especially in taxa with similar dispersal abilities. One of these well-known “suture zones” runs from NW to SE France, with a pair of pond-breeding amphibians (*Triturus cristatus/T. marmoratus* [13]) as a primary example. More recently, a study on the evolutionary history of another deeply divergent amphibian species pair (the Common toad *Bufo bufo* and the Spined toad *B. spinosus*) has revealed significant geographic concordance with the *Triturus* hybrid zone [14].

Recent molecular studies on the systematics of the *Bufo bufo* species group have resulted in the recognition of four species, two of which were formerly regarded as subspecies of *B. bufo* [14–16]. One of these, *B. spinosus,* present in Iberia and North Africa, contacts *B. bufo* in France, forming an extensive contact zone along a NW-SE line, approximately from Caen to Lyon [17]. These species split in the Late Miocene at around 9.2 MYA [14], but are morphologically similar. They can, however, be differentiated in their contact zone and recent studies have shown overall agreement between morphological characters, mitochondrial and nuclear DNA markers in delineating species boundaries in France [16]. Occasional discordance is mostly restricted to slowly evolving nuclear markers and interpreted as resulting from incomplete lineage sorting, although hybridization-mediated gene flow cannot be completely ruled out. In fact, we recently isolated and optimized a suite of 12 microsatellite loci cross-amplifying in both species and, in a preliminary assessment of patterns of gene flow across species, identified a hybrid population in a section of the contact zone in northwestern France [18]. We therefore decided to perform a more detailed analysis on a fine-scale transect across the contact zone using morphological and molecular traits (12 polymorphic microsatellites, four nuclear and two mitochondrial SNP markers) in a cline analysis framework. We here describe results on a transect across the contact zone between *B. bufo* and *B. spinosus* in northwestern France. The specific questions that we sought to answer are: i) can clines be observed for morphological, nuclear and mitochondrial markers?, ii) do clines of the different traits coincide? and iii) can the position of the contact zone be linked to features of the landscape? On a broader perspective, our goals include testing: iv) whether this contact zone is a hybrid zone, and v) whether the zone is maintained by selection, with a significant degree of reproductive isolation.

## Methods

**Fig. 1:**
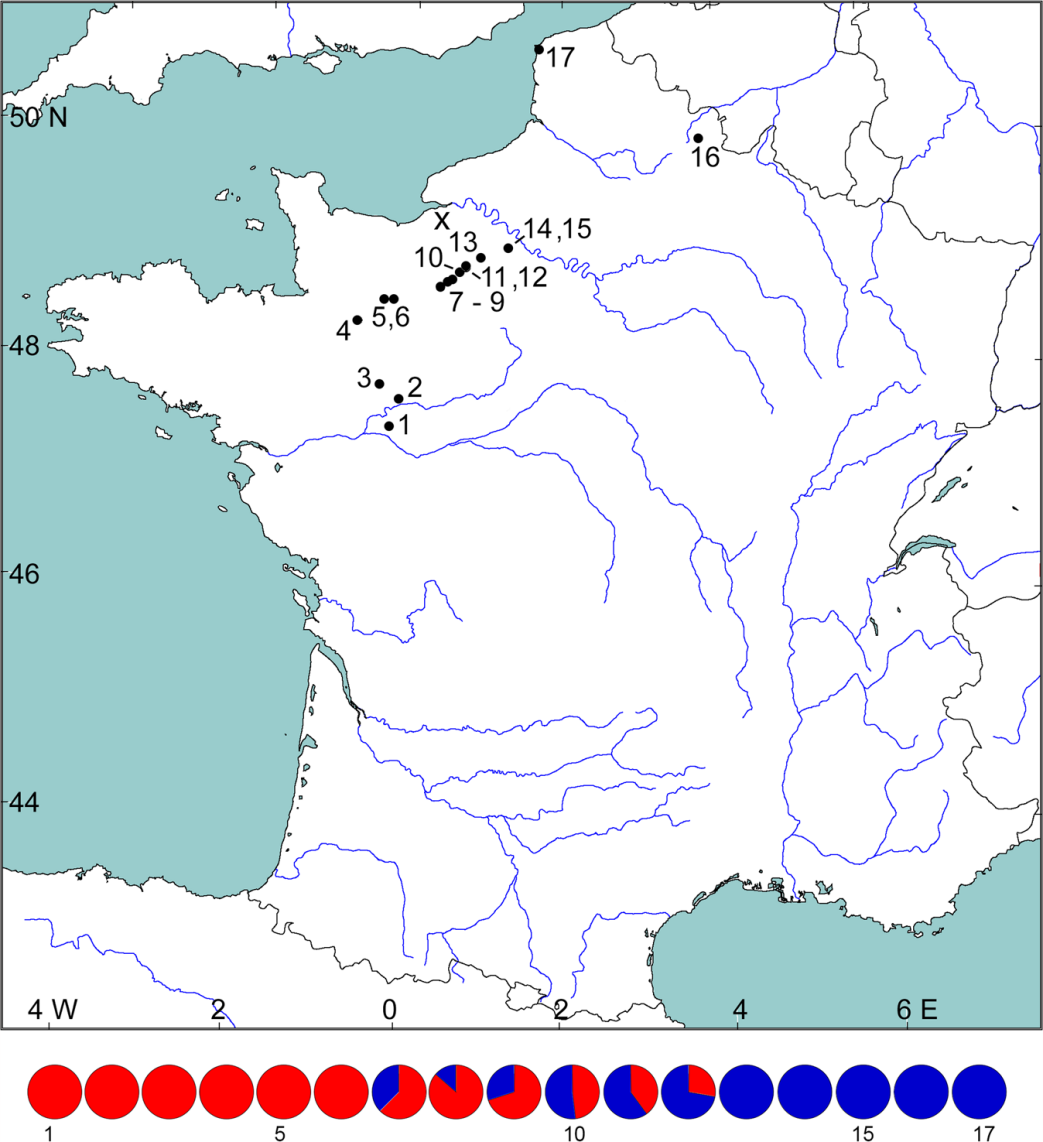
Location of 17 *Bufo spinosus* and *B. bufo* populations over a transect in northwestern France. The pie diagrams at the bottom show the observed mtDNA haplotype frequencies, with haplotypes typical for the species in red (*B. spinosus*) and blue (*B. bufo*). The *B. bufo*–*B. spinosus* hybrid population reported by [18] is shown by an X

We gathered morphological and molecular genetic data for 344 *Bufo* toads in 17 populations in a transect across the contact zone of *B. spinosus* and *B. bufo* in northwestern France (Table 1, Fig. 1) adjacent to a locality where we documented a *B. spinosus – B. bufo* hybrid population [18]. Morphological characters studied were those that help to distinguish *B. spinosus* from *B. bufo* [16], namely Snout-Urostyle length (SUl), anterior and posterior Parotoid distance (Pda, Pdp), length and width of the inner Metatarsal Tubercle (MTl, Mtw) and Paratoid angle (Pa) and the derived measures Paratoid divergence (Pd = Pda/Pdp), Metatarsal Tubercle size (MTsize = MTl/SUl) and shape (MTshape = MTw/MTl). For illustrations of these characters see [16]. Adult males (*N* = 190) and adult females (*N* = 76) were analyzed separately. For each individual we calculated the probability to belong to *B. spinosus* (P_s_), with the logistic regression formulae derived for this part of France (see [16]).

**Table 1:** Location, position in the transect and sample size for 17 *Bufo* populations included in the study. Toads studied are adults except for populations 1–3 and 5 where tadpoles were sampled

Locality		Coordinates		Altitude (m.a.s.l.) from Google Earth	Straight line distance from Beaulieu (km)	Sample size		
Population	Name	Northern latitude	Eastern longitude			Morphology (males, females)	Micro-satellites	SNPs
1	Le Poitrineau, Saumur	47.281	−0.132	65	−168.68	0	12	12
2	Étang du Perré	47.526	−0.020	92	−140.34	0	8	8
3	Les Tesnières, Durtal	47.672	−0.265	61	−134.96	0	8	8
4	Carrefour du Poteau, Jublains	48.240	−0.552	138	−107.74	27 (13, 14)	14	29
5	Les Fontaines, Pré-en-Pail	48.447	−0.201	285	−74.53	0	8	8
6	Foret de Multonne, Mont des Avaloirs	48.443	−0.132	329	−69.90	20 (18, 2)	20	20
7	Montmean, Bazoches-sur-Hoëne	48.543	0.508	210	−23.19	18 (15, 3)	31	32
8	La Couvendière, Mortagne au Perche	48.572	0.595	223	−16.34	22 (20, 2)	22	22
9	La Rosière, Foret du Perche et de la Trappe	48.600	0.639	277	−11.85	20 (18, 2)	19	20
10	Beaulieu	48.679	0.746	203	0	52 (26, 26)	40	52
11	Chateau des Bois Francs	48.719	0.832	201	7.73	2 (1, 1)	10	10
12	le Cottin	48.724	0.827	204	7.72	18 (17,1)	18	18
13	Les Quatre Vouges	48.800	1.016	175	23.95	15 (12, 3)	23	23
14	Mouettes	48.896	1.363	131	51.34	2 (2, 0)	10	10
15	Mare du Bois, La Houssaye	48.886	1.381	130	52.01	23 (22, 1)	23	23
16	Les Puits du Sarrasin, Erloy	49.915	3.847	122	264.06	25 (15, 10)	20	27
17	Les Ajoncs, Audresselles	50.821	1.602	8	246.10	22 (11, 11)	14	22
Total						266 (190, 76)	300	344
Missing data (%)						0.0	1.8	1.2

Diagnostic SNPs were developed on 16S rRNA (16S) and Cytochrome B (cytb) mitochondrial sequences from [14], yielding cytb_946 allele specific primers GGGGAGTGACTAGWGGATTTGC(G/A) and common primer ACCTCCTGGGCGACCCAGATAA and 16S_307 allele specific primers GGGTGACCACGGAGCACA(G/A) and common primer GCTTAGAAATTTTCTTTCAGCATGGAGGTT for KASP genotyping of haplotypes. For nuclear markers sequences of [14] were used to derive the following species specific SNP markers: brain-derived neurotrophic factor (BDNF) with the allele specific primers: ACTTTCTTTTTGCTATCCATGGTGAG(T/A) and common primer: TACTGGAACTCGCAATGCCGAACTA, for proopiomelanocortin (POMC) the allele specific primers: GTCATCGGAAATCTACCCAACAGA(T/G) and common primer: TCAAACTCTACAGACAACTCTCTTCTGTA, and for 60S ribosomal protein L3 (RPL3) the allele specific primers TATTCCATGACCTCATCACAGAACA(A/G) and common primer: GCAGCCMATGACTTGTGC. For the recombination activating gene 1 (RAG1) the marker sequences were from [19] with the allele specific primers: TAATCACCACAATAAATGGCCCAGA(A/G) and common primer: GGTTTAAAGTCCAAAGACCTAGAGGAATA.

KASP PCRs for both nuclear and mitochondrial SNPs were carried out in 1536 wells plates with circa 1 ng of template DNA and 1 μl of reaction mix containing KASP v4.0 reaction mix and primers following the manufacturer’s directions. The conditions of the KASP PCR were as follows: 15 min denaturation at 94 °C followed by 10 cycles of 20s at 94 °C denaturation and 60s at 61 °C of annealing and extension and lowering the annealing and extension temperature by 0.6 °C each cycle, followed by 26 cycles of 20s at 94 °C denaturation and 60s at 55 °C of annealing and extension in a hydrocycler. After 36 cycles of this touch down protocol fluorescence was measured on a Pherastar plate reader. PCR was continued and after 39, 42 and 45 cycles fluorescence was measured again to follow the trajectory of all samples. Genotypes were called automatically by the module Kraken™ of LIMS controlling the LGC genomics SNP genotyping line, visually inspected and if necessary manually corrected.

Twelve microsatellite loci were studied in 300 individuals in four multiplex reactions, following [18]. The number of alleles (Na), the observed (H_o_) and expected heterozygosity (H_e_) were calculated with GenePop 4.2 [20] per locus and population. The same program was used to test for deviations from Hardy-Weinberg equilibrium and for linkage disequilibrium, under a sequential Bonferroni correction for multiple tests [21]. For the microsatellite data the presence of null alleles was investigated with Micro-Checker 2.2.3 [22].

The software Structure [23] was used to describe population structure and identify genetically admixed individuals for SNP and microsatellite data separately. Runs consisted of 500,000 (burn-in) and 1,000,000 generations under the admixture model with correlated allele frequencies, for *K* values of 1–14, with five replicates per value of *K*. The optimal clustering scheme was selected through application of Evanno’s criterion [24] as implemented in Structure Harvester [25]. Results were processed with the pipeline CLUMPAK [26]. Individual genotypes were also assessed with NewHybrids software under default settings [27] to obtain the posterior probability that they fall into one of the following categories: *B. spinosus*, *B. bufo*, F_1_-hybrids, F_2_-hybrids and backcrosses in either direction. Structure and NewHybrids provide different but complementary information about genetic admixture and for visual inspection we constructed bivariate Structure * NewHybrid plots (see [28]).

The position, width and shape of geographical clines over the transect were determined with the R package HZAR [29] from population data for i) morphological characters (P_s_, Parotoids and Metatarsal tubercle, males and females combined), ii) mitochondrial DNA (F_s_, frequency of ‘spinosus’-haplotypes), iii) the loading on the axes of a principal component analysis derived from the panel of microsatellites (PCA1, etc.) and iv) for the nuclear genes BDNF, POMC, RAG1 and RPL3 (F_s_, the frequency of *B. spinosus* specific SNPs). As reference cline models we choose the Structure Q-score of belonging to either parental class, and the NewHybrid posterior probabilities of belonging to a hybrid class versus either of the parental species, derived from the microsatellite data. Clines are considered significantly displaced if the two log-likelihood unit support limits of the cline centre do not overlap with the Structure Q-score (Q_b_ = 1 − Q_s_). Individual microsatellite loci conveyed little information on species identity (results not shown) and GenoDive [30] was used to summarize the molecular allelic information in Principal Component vectors over all loci. The other statistical procedures were done with SPSS 21 [31].

## Results

**Fig. 2:**
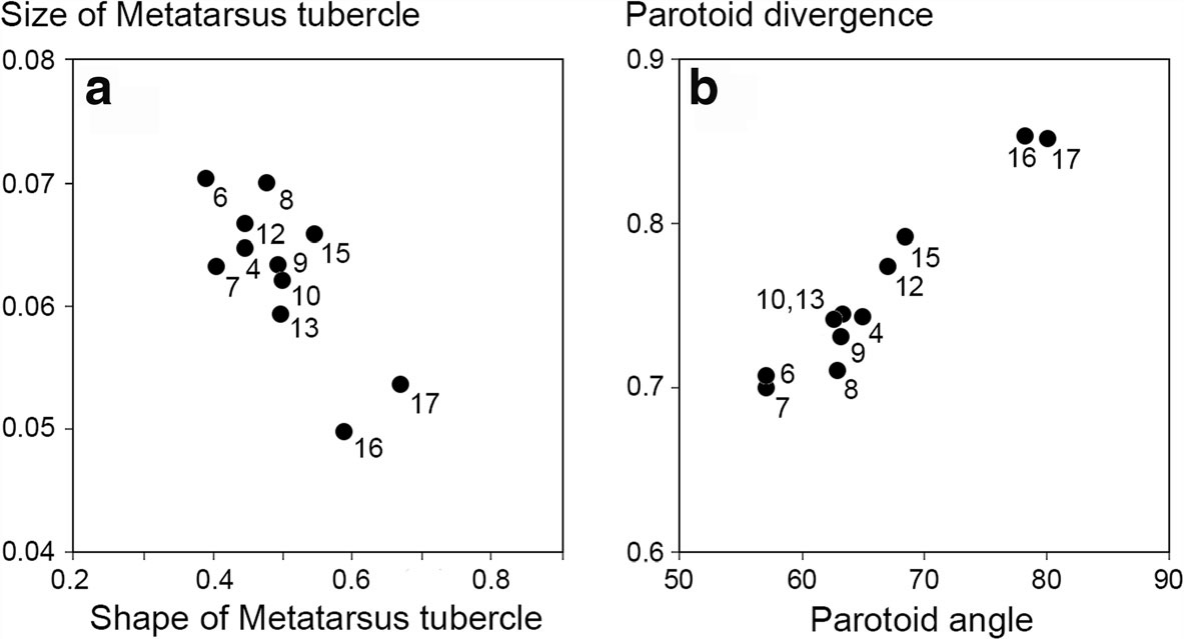
Species specific morphometric character states across a *Bufo spinosus* to *B. bufo* transect in the northwest of France. Data points shown are average values for males in 13 populations (see Table 1). **a** size and shape of the metatarsus tubercle and **b** positioning of the parotoids

At the southern side of the transect adult toads show the morphological character states typical for *B. spinosus* (metatarsus tubercle long and narrow, parotoids divergent) and at the northern side of the transect they show states typical for *B. bufo* (metatarsus tubercle small and round, parotoids parallel). Among males, morphologically intermediate individuals are most frequent in populations 9, 10, 13 and 15 (metatarsus tubercle, Fig. 2a) or in populations 12 and 15 (parotoids, Fig. 2b).

For the microsatellite data, the observed number of alleles (Na), expected (H_e_) and observed (H_o_) heterozygosities and allele size range per population are summarized in Additional file 1: Appendix I. In general, genetic diversity is higher in the centre of the transect and lower at the outer sides of the transect (Table 2A). Significant instances of linkage disequilibrium (LD) were only detected in the Bspi 3.11 × Bspi 3.19 locus combination in population 6 and in the Bspi 4.28 × Bspi 4.29 combination in population 12. Across populations LD was significant for Bs4.28 * Bs4.29 and at the centre of the transect (populations 7–12) LD was significant for Bspi4.25 * Bspi4.28 and Bpis4.27 * Bspi4.28. Micro-Checker inferred the likely presence of null alleles 12 times in nine populations. Accordingly, because of the likely presence of null alleles in loci Bspi 4.24 and 4.25, analyses with Structure and NewHybrids were repeated with these markers excluded. The results were very similar (not shown) and only results based on the full dataset are reported. Significant deviations from Hardy-Weinberg equilibrium (H_1_: heterozygote deficit) were detected eight times in seven populations, involving different loci and with no correspondence with potential null alleles (Table 2A). The percentage of variation explained along the principal component axes was 14.3, 4.7 and 4.1% for the first, second and third axis, respectively. Only the first axis was retained, since the others did not discriminate between *B. bufo* and *B. spinosus.*

**Table 2:**
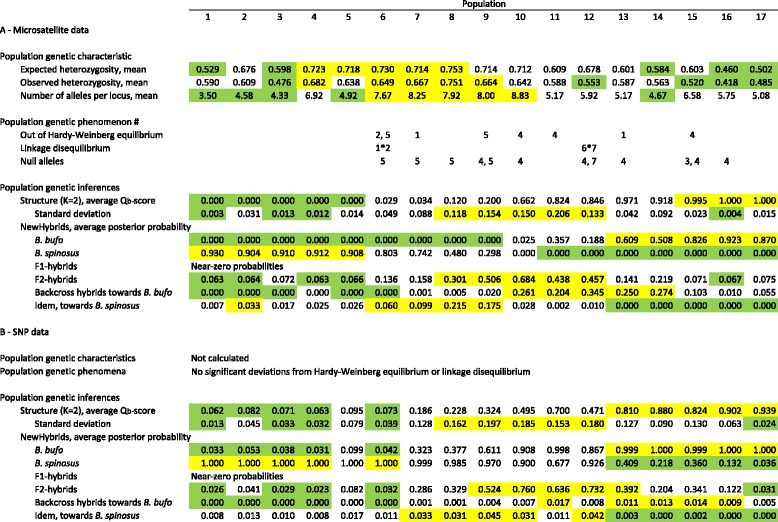
Molecular genetic characteristics across 17 populations in a *Bufo spinosus* to *B. bufo* transect in the northwest of France for 12 microsatellite loci (A) and four SNP loci (B). Within sections panels represent: upper panel–variability measures, middle panel–deviations from neutral expectations, and lower panel–proportion of populations falling into particular Structure and NewHybrids categories. Green and yellow shadings indicate values falling into the lower and upper quartiles, respectively. For locality information see Fig. 1 and Table 1. For data per locus see Additional file 1: Appendix I. The unprocessed data are in Additional file 3: Appendix III and Additional file 4: Appendix IV

^#^ Microsatellite loci are 1 - Bspi3.11, 2 - Bspi3.19, 3 - Bspi4.16, 4 - Bspi4.24, 5 - Bspi 4.25, 6 - Bspi4.28 and 7 - Bspi4.29

The number of inferred gene pools (*K*) in Structure was *K* = 2 (see Additional file 2: Appendix II), with low, intermediate and high Q_b_-scores at the southeastern, central and northwestern parts of the transect, respectively (Table 2A). In NewHybrids high values for the posterior probability of belonging to a hybrid class were observed in the central part and low scores at either side of the transect. The probability of being classified as an F_2_-hybrid was several orders of magnitude larger than for the other hybrid classes discerned, suggesting a preponderance of hybrids at deeper levels of introgressive hybridization. The results for the SNP data show the same overall pattern as that for the microsatellites, with no significant results for HW- and LD-tests (Table 2B).

**Fig. 3:**
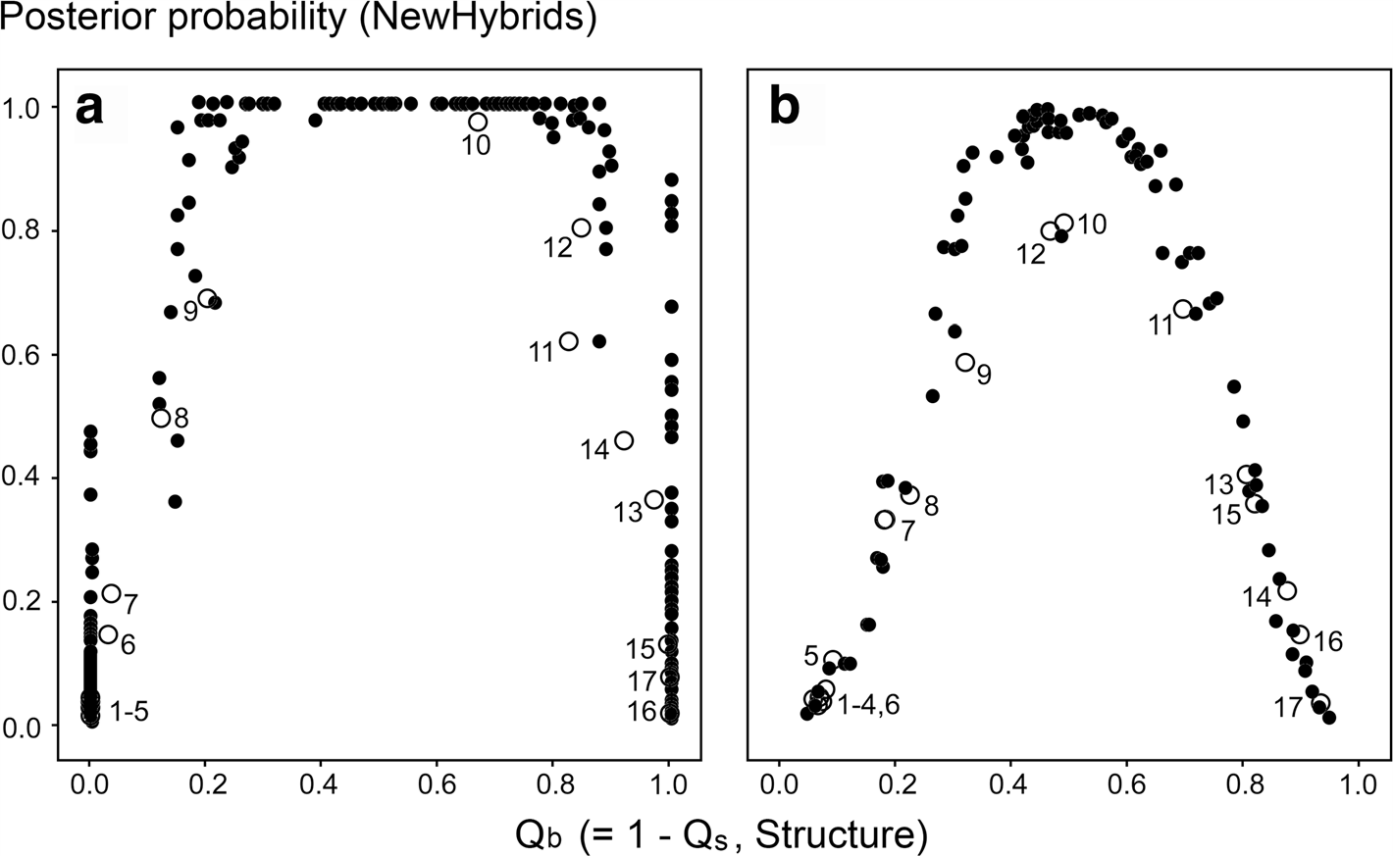
Genotype profile over 12 microsatellite markers (**a**) and four nDNA SNPs (**b**) in *Bufo spinosus* and *B. bufo* over a transect in northwestern France. The classifications are with Structure (Q-score, horizontal axis) and with NewHybrids (pp, posterior probability of falling in the pooled hybrid class, vertical axis). Data points represent individuals (*solid round symbols*) or population means (*open round symbols*). For locality information see Table 1

Bivariate Structure * NewHybrid plots show typical unimodal distributions for the microsatellite and the nuclear SNP data (Fig. 3). The bell-shaped curves suggest that the analytical results are strongly correlated between methods. Following the Structure axis, populations 1–17 are in geographical order, with few exceptions. Low hybrid values (pp < 0.2) are shown for populations 1–6 and for populations 15–17 (microsatellites) or 16 and 17 (SNPs). These populations show extreme Structure Q-scores also and represent pure or nearly pure *B. spinosus* and *B. bufo*, respectively. High hybridity values (pp > 0.8) are shown by populations 10 and 12, with intermediate values for the remaining populations. The average Q_b_-score for population 10 (Beaulieu) is 0.66 (standard deviation, SD = 0.150) suggesting that it is situated just off the centre of the contact, in the direction of *B. bufo* (see also the geographical cline analysis below). Populations 9, 11 and 12 adjacent to Beaulieu contain genetically mixed individuals with pp- and Q-scores as in Beaulieu, as well as individuals with lower pp- and intermediate Q-scores for which the status as hybrid or a parental is equivocal. The microsatellite plot (Fig. 3a) has ‘high shoulders’ (i.e. it is platykurtic), where high NewHybrids scores are coupled with extreme Structure scores. Thirty-eight out of 40 (95%) of the individuals from Beaulieu have NewHybrids scores pp > 0.975 and Structure scores 0.1 < Q < 0.9, suggesting that they have an intermediate genetic composition.

**Fig. 4:**
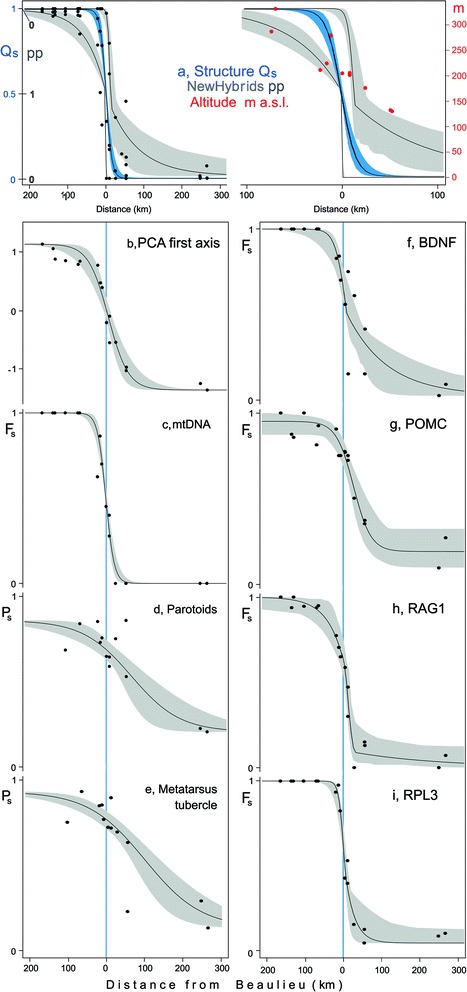
Geographical cline analysis for *Bufo spinosus* (*left*, with Q_s_-values close to unity at vertical axis) and *B. bufo* (*right*, with Q_s_-values close to zero at vertical axis) in a transect in northwestern France. Study populations are as in Table 1 and *small solid dots* are population averages. Clines were fitted to a) Structure Q-scores (with *blue shading*) and NewHybrids posterior probabilities for the species (*grey shadings* with the transition towards *B. spinosus* left and towards *B. bufo* right). Note that the graph to the *right* shows the central part of the transect in detail. Furthermore, b) the loading on the first PCA axis, based on the panel of 12 microsatellites, c) the *B. spinosus* frequency of the mtDNA marker, d, e) morphological identification probabilities (details see text) and f–i) the *B. spinosus* frequency of four SNP markers. The 95% credible cline regions are highlighted by *grey shadings*. The *vertical blue line* gives the position of population 10, at Beaulieu. Locality altitudes are plotted in *red*

A sharp cline is fitted to the Structure Q-scores for the microsatellites, accompanied by wider and less pronounced clines for the NewHybrid transitions from *B. spinosus* to the hybrid class (Fig. 4a, left hand cline) and from the hybrid class to *B. bufo* (Fig. 4a, right hand cline). In the central part of the transect, we tentatively link the position of the clines to the altitudes of the populations, that are high (≥285 m a.s.l.) for populations 5 and 6 at the *B. spinosus* side, low (≤175 m a.s.l.) for populations 13–15 at the *B. bufo* side of the transect and intermediate for the central populations 7–12 (201–277 m a.s.l.). For five out of eight characters the position of the cline is similar to that of the reference and for three out of eight also the width is similar (Table 3). Significantly displaced and significantly wider clines than the reference are observed for the two morphological characters and for the nuclear POMC locus. In all three cases the displacement is towards the *B. bufo* side of the transect (Fig. 4, Table 3). Clines wider than the reference are also found for the microsatellite information expressed in the first PCA and for the locus BDNF.

**Table 3:** Parameter estimates for the geographical clines shown in Fig. 4. The results for Structure and NewHybrids on the panel of microsatellites are taken as the reference. Significant differences with the 2log-likelihood unit support limits falling outside the range observed for the reference clines are shown in boldface type

	Model								
Character, unit	Type	Centre	Width	*δ*_*L*_	*δ*_*R*_	*τ*_*L*_	*τ*_*R*_	*p*_*min*_	*p*_*max*_
Microsatellite reference clines									
Structure, Q	fixN	−1.0 (−1.0–0.7)	30.3 (21.5–44.4)					0.016	0.983
NewHybrids, posterior probability									
*- B. spinosus* side of transect	fixL	−1.0 (−1.0–−0.2)	0.6 (0.0–1.3)	0.000		0.007		0.000	0.990
*- B. bufo* side of transect	typR	10.8 (8.4–20.0)	12.2 (6.0–29.9)		1.729		0.048	0	1
Thresholds–min, max		−1.0, 20.0	0.0, 44.4						
Tested clines									
PCA on microsatellites, loading first axis	fixN	4.6 (−1.0−15.7)	**104.9 (64.4–179.7)**					−1.301	1.156
mtDNA, haplotype frequency	typN	−1.0 (−1.0–2.4)	43.5 (31.0–64.0)					0	1
Parotoids, classification probability	fixN	**65.5 (27.3–122.8)**	**255.1 (107.7–> 400)**					0.142	0.769
Metatarsal tubercle, idem	fixN	**96.6 (54.2–145.3)**	**298.7 (164.4–> 400)**					0.060	0.865
BDNF, allele frequency	fixR	3.8 (−0.9–18.6)	**58.9 (46.9–115.8)**		0.505		0.274	0.023	1.000
POMC, idem	optN	**22.3 (9.2–41.5)**	**90.7 (52.6–176.7)**					0.187	0.951
RAG1, idem	typB	4.0 (−0.7–9.1)	35.7 (23.4–56.2)	2.811	20.704	0.360	0.042	0	1
RPL3, idem	fixR	−0.9 (−1.0–3.9)	26.8 (23.0–49.1)		1.064		0.507	0.050	1.000

For the chosen models *p*_*min*_ and *p*_*max*_ are fixed to 0 and 1 (typ models), or their empirical values (fix models), or *p*_*min*_ and *p*_*max*_ are fitted (opt model). Tail fitting encompassed right (*R*), left (*L*), none (*N*) or both fitted (*B*). Cline position is the centre of the contact zone and ca. equals the distance to Beaulieu (in km, population 10, see Table 1). Cline width (w) is calculated as 1/maximum slope. Two log-likelihood unit support limits are presented in parentheses. δ and τ are the shape parameters for the left and right tails, and *p*_*min*_ and *p*_*max*_ are the character states at either end of the transect

## Discussion

Secondary hybrid zones form when taxa that have evolved in allopatry meet and exchange genes. The outcome of the species interaction depends on the evolution of mechanisms of reproductive isolation, both pre- and post-zygotic, in which isolation usually increases with time since divergence, with examples all along the continuum between genetic merger (as in ‘hybrid species’) and full reproductive isolation. For amphibian examples in which the extent of natural hybridization in contact zones correlates with divergence times see refs. [28, 32, 33].

In the hybrid zone studied here, most hybrid individuals were classified by NewHybrids as F_2_s or backcrosses. While this could represent differences in fertility between hybrid classes, it is also likely that later generation hybrids and backcrosses are being pooled together in the F_2_ class, since the confidence of assignment to different hybrid classes depends on the number of generations elapsed after secondary contact [34] and the contact is probably old (see below). The lack of full reproductive isolation may seem surprising given that our study system is formed by two non-sister toad species that diverged in the Miocene [14, 35]. Thus, some degree of reproductive isolation due to the evolution of genetic incompatibilities was expected, but long lasting genetic compatibility is not uncommon in amphibians (e.g. [28]). Initial assessments of patterns of allele sharing across species in this system were inconclusive as to whether gene flow or incomplete lineage sorting best explained observed discordance between mtDNA and nDNA data [14, 16, 17, 19]. This, and the characterization of a hybrid population based on morphological and molecular genetic data [18], raised questions about the extent of reproductive isolation in this species pair. However, our comprehensive transect sampling has revealed sharp, spatially concordant and narrow genetic clines (Fig. 4), suggestive of barriers to hybridization. Tentatively, this hybrid zone can be located along the ‘Collines de Normandie’, with *B. spinosus* at higher altitudes than *B. bufo*, and a centre close to locality 10 (Beaulieu) in our transect. Nevertheless, it is unclear the extent to which selection falls on the parentals and/or the hybrids and whether environmentally mediated selection plays a role in stabilizing the hybrid zone. The taxonomic status of *B. spinosus* as a species different from *B. bufo* is recent [14, 16, 18, 19, 36–39] and not entirely without debate [17, 35]. The results showing limited gene flow across a narrow hybrid zone (despite no barriers to dispersal) bring evidence that reproductive isolation is probably involved and further argues for species status of the Spined toad, *B. spinosus*.

In general, allopatric populations of the two parental species have lower genetic diversity than populations near the centre of the contact zone. The inspection of allele frequencies shows a clear trend in some loci, with some alleles presenting high frequencies on opposite sides of the contact zone and intermediate frequencies co-occurring in the centre of the contact zone. Genetic homogenization due to hybridization is spatially restricted. This is also apparent upon inspection of Structure and NewHybrids analyses, which show increasing levels of admixture close to the centre of the contact zone. Thus, while we have found evidence for ongoing hybridization between the two *Bufo* species, evidence for hybrids is restricted to a narrow area about 30 km wide. Most hybrids were classified as F_2_ individuals by NewHybrids, which is often the case in relatively old contact zones where many generations of backcrossing complicate precise assignment of individuals to specific hybrid classes [8, 18, 33, 34]. While the original position of this hybrid zone is unknown, the default scenario is that it formed at or close to its current location within the last glacial cycle.

Phylogeographic analyses on the basis of two different climate reconstructions for the Last Glacial Maximum (LGM), MIROC and CCSM, lead to drastically different scenarios [35]. Under MIROC, climate conditions are favourable over most of Europe south of 50°N, therewith suggesting that the *B. bufo* - *B. spinosus* contact pre-dates the LGM. Conversely, under CCSM favourable areas are situated at more southern latitudes, with small fragments in coastal areas (both at the Atlantic and the Mediterranean) and large continuous areas restricted to southern Europe, suggesting that the *Bufo* hybrid zone formed more recently. We propose a scenario in which *B. spinosus* reached the Normandy region around 8000 YBP, because the species is absent from the Channel island of Guernsey that became isolated from the continent shortly before that date, but made it to Jersey [19]. Jersey was the last of the Channel Islands to be separated from the European mainland, with the final inundation of the English Channel in roughly that period [40]. In parallel and over the same period *B. bufo*, with a glacial refugium in the Balkan peninsula, reached the landmass that is currently England, Scotland and Wales, but it didn’t reach the Isle of Man or Ireland. We therefore assert that the species contact is thousands of years old, but further studies are required to reconstruct the species’ postglacial dynamics and to track the spatial and temporal stability of the hybrid zone.

The concordance of mtDNA and nuclear clines is expected when there is selection against F_1_ hybrids, but sex-biased dispersal or asymmetric genetic incompatibilities among the sexes can produce discordant clines. While data for *B. spinosus* are scarce, a study on British and Swedish populations of *B. bufo* found that 93% of females and 96% of males that survived between years returned to the same breeding ponds [41], together with direct observations [42] suggesting the absence of sex biased dispersal. Furthermore, a cline as narrow as observed cannot result from neutral processes exclusively. In the absence of selection, the width (*w*) of the cline can be predicted from a diffusion model as a function of dispersal distance (*d*) and the length of time since contact (*t*), as *w* = 2.51 *d* √*t* [43]. The maximum dispersal distance recorded for *B. bufo*/*B. spinosus* averages at 1.32 km in eight studies (range 0.12–3.62 km) [42, 44, 45] Generation time is 2 years for male and 3 years for female *Bufo* [46]. At a low average dispersal of 1 km per generation, cline widths would exceed the 30.3 km width of the Structure Q reference cline (Table 3) in ca. 400 years and at a high average dispersal of 5 km per generation this would last only 17 years. Arguably, the hybrid zone is much older than this and cline width appears to have been kept in check over several thousands of years, presumably through selection against hybrids, especially F_1_’s, and/or the parentals, given their absence and low frequency at the centre of the zone, respectively.

Despite the overall congruence of genetic clines, with similar patterns between nuclear and mitochondrial markers, some clines show smoother transitions between pure populations at both ends of the transect. One remarkable discrepancy is between the sharp species transition documented by the Structure and NewHybrid analyses of the microsatellite data (Fig. 4A) versus the smooth transition revealed by the first PCA-axis obtained from same data (Fig. 4B). Given the general evidence for a narrow, ca. 30 km wide hybrid zone, this result suggests that PCA does not perform well in extracting the species-specific character states. Also the morphological clines are smooth (Fig. 4DE), which can be due to a variety of factors, all of which are worth further exploration. Possibly, some species diagnostic morphological character states are yet to be discovered. On the other hand, morphological features distinguishing the two toad species are probably the result of additive effects from many genes and complex interactions with the environment, so perhaps a looser connection than with SNP or microsatellite loci should be expected. Finally, sampling variance or micro-environmental selection could also have a role in smoothing the morphological cline. Whatever the case, the presumably extensive contact zone offers ample opportunities to test the consistency of previously identified morphological and genetic characters in diagnosing species across different sections of their contact zone.

## Conclusion

We have characterized the common toad system as a hybrid zone, with narrow and coincident clines revealing barriers to gene flow across species, which opens new and exciting lines of research to further pursue with the help of genomic tools. While the relative roles of intrinsic and extrinsic factors in shaping genetic and morphological clines are still open to further scrutiny, the geographical extent of this contact zone, encompassing France from the northwest to the southeast, allows the comparative study of multiple transects, with great opportunity for the identification of consistently versus locally-introgressed genomic regions and thus for discerning the relative roles of indeterminate drift and selection in the origin and maintenance of species boundaries. Thus, we assert that the hybrid zone between *B. bufo* and *B. spinosus* will become a prime study system in speciation research.

## Additional files

Additional file 1
**Appendix I.** Genetic polymorphisms in a transect across 17 *Bufo spinosus* and *B. bufo* populations in northwestern France, estimated for 12 microsatellite loci: Na–number of alleles, H_o_–observed heterozygosity, H_e_–expected heterozygosity and the size range of alleles in base pairs. Data for populations 1–3, 16 and 17 are from [18]. (XLSX 17 kb)
Additional file 2
**Appendix II.** Result of Structure analysis, showing the deltaK graph towards the selection of *K* = 2. (TIF 49 kb)
Additional file 3
**Appendix III.** Microsatellite data in GenePop format [20]. Populations are in the order 1–17, as in Table 1. (TXT 27 kb)
Additional file 4
**Appendix IV.** SNP data in GenePop format [20]. Populations are in the order 1–17, as in Table 1. (TXT 9 kb)
