# Supplementary figures and images for: Concordant morphological and molecular clines in a contact zone of the Common and Spined toad (Bufo bufo and B. spinosus) in the northwest of France
Source: Front Zool. 2016 Dec 19;13:52. doi: 10.1186/s12983-016-0184-7 (PMC5168812; doi:10.1186/s12983-016-0184-7)

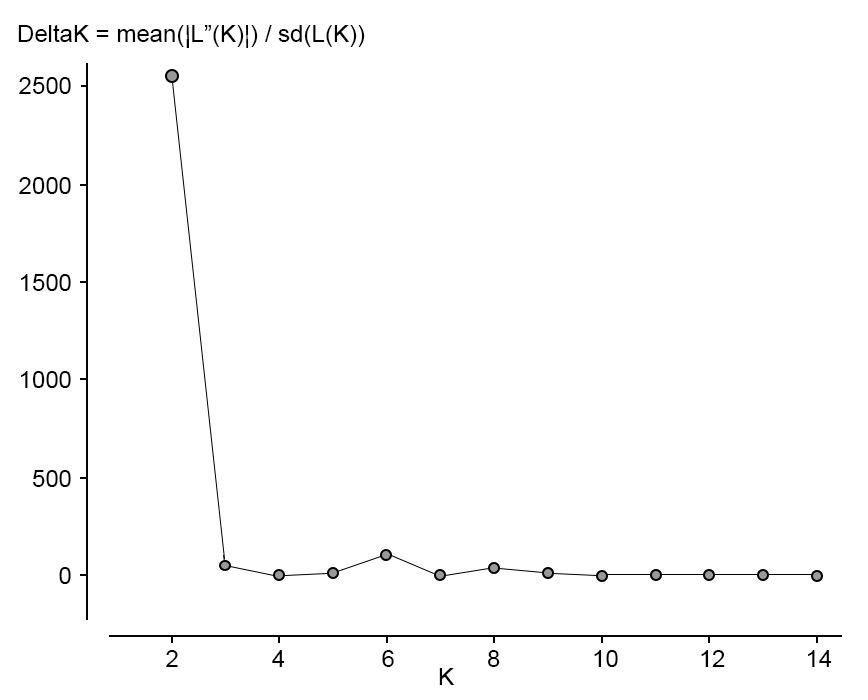

Supplement: Additional file 2 — Appendix II. Result of Structure analysis, showing the deltaK graph towards the selection of K = 2. (TIF 49 kb) [file 12983_2016_184_MOESM2_ESM.tif]
